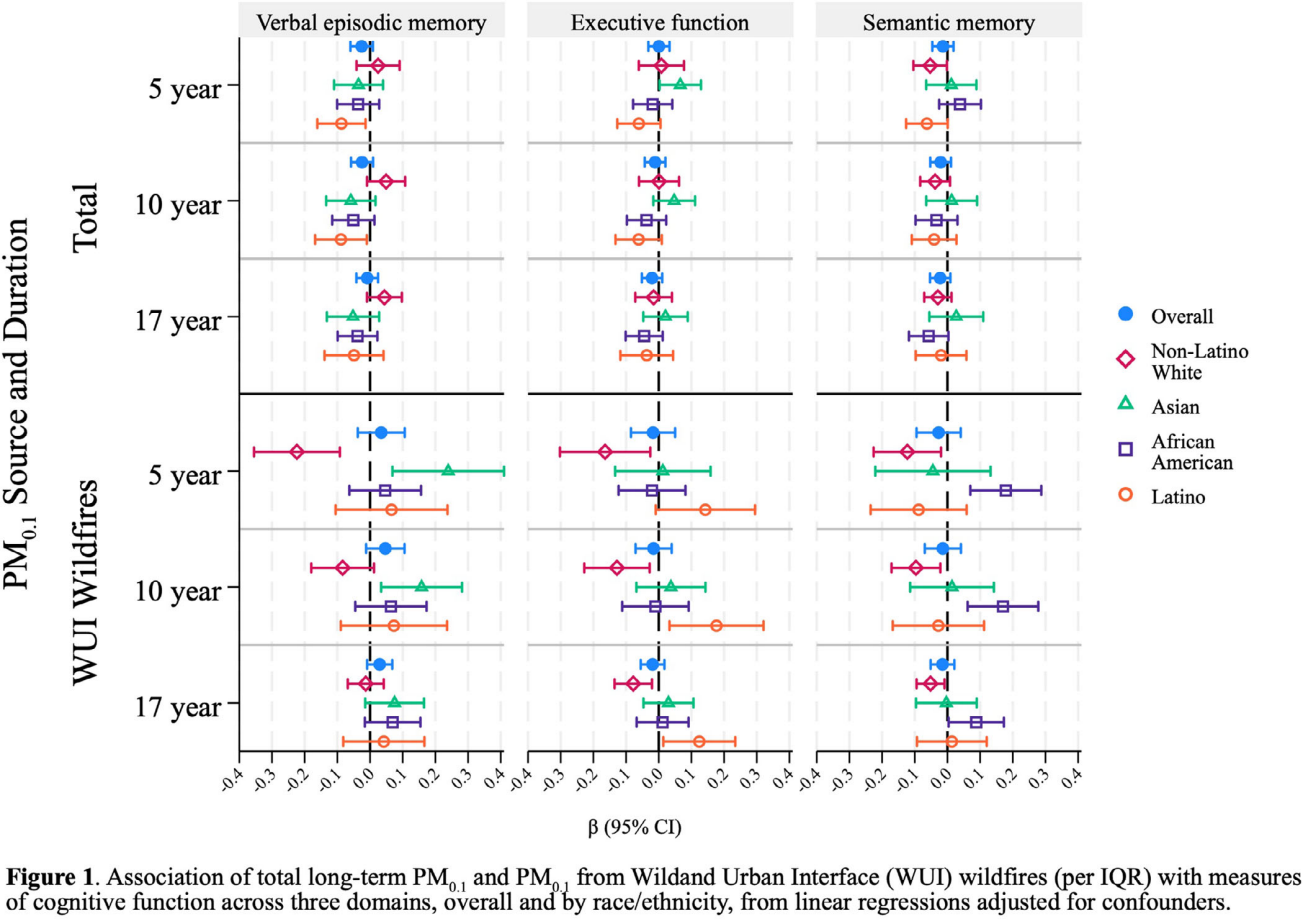# Ultrafine particulate air pollution from wildfires and late‐life cognitive function in the KHANDLE and STAR cohorts

**DOI:** 10.1002/alz70860_103903

**Published:** 2025-12-23

**Authors:** Kathryn C Conlon, Amanda Goodrich, Stacey Alexeeff, Ai‐Lin Tsai, Paola Gilsanz, Mike Kleeman, Rachel A. Whitmer

**Affiliations:** ^1^ University of California, Davis, Davis, CA, USA; ^2^ University of California Davis, Davis, CA, USA; ^3^ Kaiser Permanente Northern California Division of Research, Pleasanton, CA, USA; ^4^ UC Davis, Davis, CA, USA

## Abstract

**Background:**

Substantial research has linked fine particulate matter (PM<2.5 μm in diameter, PM_2.5_) to cardiovascular disease and dementia, however, the role of ultrafine particulate matter (PM<0.1 μm in diameter, PM_0.1_), which is more readily absorbed into the bloodstream due to its smaller size, remains underexplored, especially in diverse populations.

**Method:**

The study included data from *n* = 1648 adults aged ≥65 in the ethnically diverse Kaiser Healthy Aging and Diverse Life Experiences (KHANDLE) study, and *n* = 746 African Americans in the Study of Healthy Aging in African‐Americans (STAR). Three domains of cognitive outcomes (semantic memory, verbal episodic memory, and executive function were measured using the Spanish and English Neuropsychological Assessment Scales and Z‐scored. Total PM_0.1_, and the fraction of PM_0.1_ from two sources (biomass combustion and wildland urban interface (WUI)), during 2000‐2020 were generated using a chemical transport model and linked to geocoded residential addresses. We used linear regression to assess the association between long‐term exposure to PM_0.1_ (5‐, 10‐, and 17‐year averages) and domain‐specific cognitive function adjusting for age, self‐reported gender, education (≤High School, Trade/College, and Graduate School), marital status (married/living as married, not married, missing), neighborhood income, and study. Effect modification by race/ethnicity was assessed using a multiplicative term, with *p* < 0.05 indicating significant effect modification.

**Result:**

No associations were observed in main effects models (Figure 1). Effect modification by race and ethnicity was observed with 5‐year WUI PM_0.1_ exposures across all cognitive domains (*p* < 0.05). Among non‐Latino white participants, an IQR increase in 5‐year average WUI PM_0.1_ was associated with a decrease in cognition across all cognitive domains (Figure 1). Among Latino participants, an IQR increase in 5‐year average total PM_0.1_ was associated with decreased verbal episodic memory. Associations were attenuated or null for 10‐ and 17‐year exposures.

**Conclusion:**

Long‐term WUI PM_0.1_ exposure was associated with decreased cognitive function among non‐Latino white participants. Wildfires are an increasing threat in California, and more research on their impact on cognition is needed.